# Efficacy and Safety of SGLT2 Inhibitors and Sotagliflozin in Heart Failure: A Systematic Review and Meta-Analysis of 59 Randomized Controlled Trials

**DOI:** 10.7759/cureus.113025

**Published:** 2026-07-20

**Authors:** Vicky Muller Ferreira, Victor Ayres Muller

**Affiliations:** 1 Cardiology, Independent Researcher, Rio de Janeiro, BRA; 2 Internal Medicine, Hospital Universitário de Vassouras, Rio de Janeiro, BRA

**Keywords:** all-cause mortality, gliflozin, heart failure, heart failure hospitalization, meta-analysis, sglt2 inhibitors, systematic review

## Abstract

Sodium-glucose cotransporter-2 (SGLT2) inhibitors have become a cornerstone of heart failure (HF) therapy, yet the totality of randomized evidence - including smaller trials beyond the landmark cardiovascular (CV) outcome studies - has not been comprehensively synthesized. We searched PubMed, Cochrane Central Register of Controlled Trials (CENTRAL), ClinicalTrials.gov, and the World Health Organization International Clinical Trials Registry Platform (ICTRP) from inception to March 2026 for randomized controlled trials (RCTs) comparing any SGLT2 inhibitor or the dual sodium-glucose cotransporter-1/2 (SGLT1/SGLT2) inhibitor sotagliflozin with placebo or standard care in adults with HF. Random-effects Mantel-Haenszel risk ratios (RRs) with Hartung-Knapp-Sidik-Jonkman (HKSJ) confidence intervals (CIs) were used. Certainty of evidence was assessed using the Grading of Recommendations Assessment, Development and Evaluation (GRADE) approach. The protocol was prospectively registered with the International Prospective Register of Systematic Reviews (PROSPERO; CRD420251167908). Of 6,239 records identified, 114 studies met inclusion criteria and 59 RCTs (29,692 participants) were included in the quantitative synthesis. SGLT2 inhibitors reduced all-cause mortality (ACM; RR 0.90, 95% CI 0.83-0.98; p = 0.016; 26 trials; low certainty), although trim-and-fill adjustment for publication bias attenuated the finding (adjusted RR 0.92, 95% CI 0.84-1.00). HF hospitalization (HFH) was reduced (RR 0.74, 95% CI 0.69-0.79; 15 trials; moderate certainty), as were CV death (RR 0.86, 95% CI 0.76-0.98; seven trials; moderate certainty), the composite of CV death and HFH (RR 0.80, 95% CI 0.75-0.85; eight trials; high certainty), and serious adverse events (RR 0.94, 95% CI 0.90-0.99; 20 trials; high certainty). Genital infections were significantly increased (RR 3.75, 95% CI 1.72-8.19); no significant increases were observed for diabetic ketoacidosis (DKA), acute kidney injury (AKI), urinary tract infection (UTI), or hypotension. Subgroup analyses showed a greater ACM benefit in HF with reduced ejection fraction (HFrEF) than in HF with preserved ejection fraction (HFpEF) (interaction p = 0.039). Results were directionally consistent across 13 sensitivity scenarios and five alternative statistical models. SGLT2 inhibitors reduce HFH, CV death, the composite endpoint, and serious adverse events with moderate-to-high certainty; the all-cause mortality reduction is of low certainty and sensitive to publication bias.

## Introduction and background

Heart failure (HF) affects over 64 million people worldwide and remains a leading cause of hospitalization and death, with five-year mortality rates approaching 50% despite advances in pharmacotherapy [1]. The economic burden is substantial, with global costs exceeding 100 billion United States dollars annually, driven largely by recurrent hospitalizations [1]. Although guideline-directed medical therapy, including renin-angiotensin-aldosterone system inhibitors, beta-blockers, and mineralocorticoid receptor antagonists, has improved outcomes in HF with reduced ejection fraction (HFrEF), progress in HF with preserved ejection fraction (HFpEF) has been more limited until recently [2-3].

Sodium-glucose cotransporter-2 (SGLT2) inhibitors, originally developed as glucose-lowering agents for type 2 diabetes mellitus, have demonstrated unexpected cardiovascular (CV) benefits that extend beyond glycemic control. The DAPA-HF and EMPEROR-Reduced trials established dapagliflozin and empagliflozin as effective therapies for HFrEF [4-5], while DELIVER and EMPEROR-Preserved expanded these benefits to HFpEF [6-7]. These landmark trials led to the inclusion of SGLT2 inhibitors as a foundational pillar of HF therapy in both the 2022 American Heart Association/American College of Cardiology/Heart Failure Society of America (AHA/ACC/HFSA) and 2023 European Society of Cardiology (ESC) guidelines, applicable across ejection fraction categories [2-3].

Several meta-analyses have synthesized evidence from the major SGLT2 inhibitor trials in HF [8-9]. However, most have focused on a limited number of large, pivotal trials and have not incorporated the growing body of smaller randomized controlled trials (RCTs) that collectively enroll thousands of patients and provide additional data on safety outcomes, quality-of-life measures, and specific patient populations. Furthermore, the landscape continues to evolve, with new trials published through early 2026 that have not been captured in prior syntheses.

The objective of this systematic review and meta-analysis was to comprehensively evaluate the efficacy and safety of SGLT2 inhibitors compared with placebo or standard care in adults with HF, incorporating all available RCTs regardless of sample size, drug, or HF subtype. We aimed to (1) estimate the pooled effects on all-cause mortality (ACM), HF hospitalization (HFH), CV death, and other clinical endpoints; (2) explore sources of heterogeneity through subgroup analyses; and (3) assess the certainty of evidence using the Grading of Recommendations Assessment, Development and Evaluation (GRADE) approach.

## Review

Methodology

Protocol and Registration

This systematic review was conducted in accordance with the Preferred Reporting Items for Systematic Reviews and Meta-Analyses (PRISMA) 2020 guidelines and was prospectively registered with the International Prospective Register of Systematic Reviews (PROSPERO; CRD420251167908) [10-11]. Five amendments to the PROSPERO record document the search restart (March 2026), exclusion of LILACS (portal inaccessible during execution), exclusion of Embase (no institutional access), disclosure of artificial intelligence (AI) tool use for screening triage and extraction structuring, and clarification of the dual sodium-glucose cotransporter-1/2 (SGLT1/SGLT2) inhibitor sotagliflozin within the intervention scope. The protocol with all amendments is provided as supplementary material on Zenodo.

Eligibility Criteria

We included RCTs that enrolled adults (age 18 years or older) with a clinical diagnosis of HF of any phenotype - HF with reduced ejection fraction (HFrEF; left ventricular ejection fraction [LVEF] less than 40%), HF with mildly reduced ejection fraction (HFmrEF; LVEF 40-49%), HF with preserved ejection fraction (HFpEF; LVEF 50% or greater), or unspecified - and randomized participants to any sodium-glucose cotransporter-2 (SGLT2) inhibitor (empagliflozin, dapagliflozin, canagliflozin, ertugliflozin, ipragliflozin, luseogliflozin, tofogliflozin, remogliflozin, bexagliflozin, henagliflozin, or licogliflozin) or the dual SGLT1/SGLT2 inhibitor sotagliflozin versus placebo or standard care. Sotagliflozin was included because it shares the SGLT2 inhibitory mechanism and has been evaluated in HF-specific trials (SOLOIST-WHF [12]); its inclusion is examined in a pre-specified sensitivity analysis. We excluded observational studies, single-arm trials, studies enrolling exclusively pediatric populations, studies with active comparators (head-to-head drug comparisons), and sub-analyses of already-included trials. No language or date restrictions were applied. The full Population, Intervention, Comparator, Outcomes, and Study Design eligibility criteria are summarized in Table 1.

**Table 1:** Eligibility criteria (PICOS) Population, Intervention, Comparator, Outcomes, and Study Design eligibility criteria applied during full-text screening.

Domain	Inclusion	Exclusion
Population	Adults (>=18 years) with clinical diagnosis of heart failure of any phenotype (HFrEF [LVEF <40%], HFmrEF [LVEF 40-49%], HFpEF [LVEF >=50%], or unspecified)	Pediatric populations (<18 years); studies enrolling exclusively patients with type 2 diabetes mellitus and incidental heart failure (no HF inclusion criterion)
Intervention	Any SGLT2 inhibitor (empagliflozin, dapagliflozin, canagliflozin, ertugliflozin, ipragliflozin, luseogliflozin, tofogliflozin, remogliflozin, bexagliflozin, henagliflozin, licogliflozin) OR the dual SGLT1/SGLT2 inhibitor sotagliflozin	Non-SGLT2 interventions; combination products without SGLT2 inhibitor isolated as a study arm
Comparator	Placebo OR standard care (usual care without SGLT2 inhibitor)	Active drug-vs-drug comparisons (head-to-head between two SGLT2 inhibitors or vs another HF therapy)
Outcomes	At least one of: all-cause mortality, HF hospitalization, cardiovascular death, composite of CV death and HFH, serious adverse events, KCCQ total or overall summary score, six-minute walk distance, NT-proBNP, eGFR, body weight, systolic blood pressure, or specified safety outcomes (DKA, AKI, UTI, hypotension, genital infection)	Studies reporting only surrogate biomarkers without any clinical or patient-reported outcome
Study design	Randomized controlled trials (parallel-group or cluster RCT with design-effect adjustment)	Observational studies; single-arm trials; sub-analyses of already-included trials; conference abstracts without sufficient outcome data; protocols or registry records without published results
Other	No language restrictions; no date restrictions	Duplicate publications of the same trial population (most recent and most population-comprehensive primary publication retained)

Information Sources and Search Strategy

We searched PubMed, Cochrane Central Register of Controlled Trials (CENTRAL), ClinicalTrials.gov, and the World Health Organization International Clinical Trials Registry Platform (ICTRP) from inception through March 3, 2026. The original protocol also specified LILACS as a data source; the LILACS portal returned HTTP 403 errors during execution and was inaccessible. Scoping searches conducted before LILACS became inaccessible identified no SGLT2 inhibitor HF RCTs published exclusively in LILACS-indexed journals not already captured by the other databases. The deviation was registered as a PROSPERO amendment. Embase was not searched due to lack of institutional access for the independent research team (full database-sensitivity rationale for both omissions in Appendix A). The search strategy combined three concept blocks: SGLT2 inhibitors (Medical Subject Headings [MeSH] terms, pharmacological action terms, and free-text synonyms for all individual drugs plus the class wildcard "gliflozin*"), HF (MeSH terms and free-text variants), and study design (RCT filter for PubMed; no design filter for CENTRAL or trial registries). Citation chasing of included studies and prior meta-analyses was used to mitigate the risk of missed records. The full search strings, records retrieved, and execution dates for the four databases formally searched are summarized in Table 2.

**Table 2:** Search strategies and yields by database Database, full search string, records retrieved, and date of execution. Original protocol also specified Latin American and Caribbean Health Sciences Literature (LILACS); the database was inaccessible during execution and the deviation was registered as a PROSPERO amendment. Embase was not searched due to lack of institutional access.

Database	Search Strategy	Records Retrieved	Date
PubMed	("Sodium-Glucose Transporter 2 Inhibitors"[MeSH] OR "Sodium-Glucose Transporter 2 Inhibitors"[Pharmacological Action] OR "SGLT2 inhibitor*"[tiab] OR "SGLT-2 inhibitor*"[tiab] OR "sodium-glucose cotransporter 2 inhibitor*"[tiab] OR "gliflozin*"[tiab] OR empagliflozin[tiab] OR dapagliflozin[tiab] OR canagliflozin[tiab] OR sotagliflozin[tiab] OR ertugliflozin[tiab] OR ipragliflozin[tiab] OR luseogliflozin[tiab] OR tofogliflozin[tiab] OR remogliflozin[tiab] OR bexagliflozin[tiab] OR henagliflozin[tiab] OR licogliflozin[tiab]) AND ("Heart Failure"[MeSH] OR "heart failure"[tiab] OR "cardiac failure"[tiab] OR "congestive heart failure"[tiab] OR HFrEF[tiab] OR HFpEF[tiab] OR HFmrEF[tiab] OR "preserved ejection fraction"[tiab] OR "reduced ejection fraction"[tiab] OR "mildly reduced ejection fraction"[tiab] OR "ventricular dysfunction"[tiab]) AND (Randomized Controlled Trial[pt] OR "randomized"[tiab] OR "randomised"[tiab] OR "randomly"[tiab] OR "trial"[tiab] OR "groups"[tiab])	2243	2026-03-03
Cochrane CENTRAL	#1 [mh "Sodium-Glucose Transporter 2 Inhibitors"] OR #2 ((SGLT2 NEXT inhibitor*) OR gliflozin* OR empagliflozin OR dapagliflozin OR canagliflozin OR sotagliflozin OR ertugliflozin OR ipragliflozin OR luseogliflozin OR tofogliflozin OR remogliflozin OR bexagliflozin OR henagliflozin OR licogliflozin):ti,ab,kw AND #4 [mh "Heart Failure"] OR #5 ("heart failure" OR "cardiac failure" OR HFrEF OR HFpEF OR HFmrEF OR "ventricular dysfunction"):ti,ab,kw | Filter: CENTRAL only	2064	2026-03-03
ClinicalTrials.gov	Condition: Heart Failure | Intervention: SGLT2 inhibitor OR empagliflozin OR dapagliflozin OR canagliflozin OR sotagliflozin OR ertugliflozin OR ipragliflozin OR luseogliflozin OR tofogliflozin OR remogliflozin OR bexagliflozin OR henagliflozin OR licogliflozin OR gliflozin | Study Type: Interventional (Clinical Trial)	1637	2026-03-03
WHO ICTRP	Title: SGLT2 AND heart failure; Title: gliflozin AND heart failure; Condition: heart failure (deduplicated across the two title queries)	295	2026-03-03
LILACS	Not searched. Original protocol specified LILACS but the portal (pesquisa.bvsalud.org) returned HTTP 403 errors during execution. Deviation registered as PROSPERO amendment.	NA	2026-03-03
Embase	Not searched. No institutional access for the independent research team. Deviation registered as PROSPERO amendment.	NA	NA
Total identified	Sum across formally searched databases	6239	2026-03-03

Study Selection

Records were managed through a multi-phase screening process by two independent reviewers (VMF, VAM). After automated deduplication using exact identifier matching (PubMed identifier (PMID), digital object identifier (DOI), ClinicalTrials.gov identifier (NCT)) and fuzzy title matching (95% threshold), title and abstract screening was performed using a four-tier exclusion hierarchy (animal/in vitro, wrong intervention, wrong population, and wrong design), with key trial protection to prevent false exclusion of sentinel studies. Initial title and abstract classification was facilitated by a large language model (Claude, Anthropic) to flag records as likely eligible, likely ineligible, or uncertain; all flagged records and all uncertain classifications were independently verified by both reviewers. Disagreements were resolved by consensus. Full-text screening used a structured multi-pass pipeline incorporating automated eligibility checks, application programming interface-based metadata enrichment (PubMed, ClinicalTrials.gov), trial clustering to identify sub-analyses and companion publications, independent reviewer assessment in blocks of 25 records, and full-text retrieval with Population/Intervention/Comparator/Outcome (PICO) verification. A detailed description of each screening pass, including decision rules, is provided in Appendix B.

Data Extraction

Data were extracted from full-text PDFs of included studies using a standardized form with approximately 175 fields. Initial structured extraction was performed using large language models (Claude, Anthropic; Codex, OpenAI) to parse PDF content into a standardized long-format structure (record identifier, field name, and value); all extracted values were independently verified against source PDFs by both reviewers (VMF and VAM), with discrepancies resolved by consensus and reference to the original publication. AI tools were used in a triage and structuring role only; AI was not used for risk of bias (ROB) assessment, statistical analysis, GRADE certainty rating, or interpretation (full AI-use disclosure in Appendix C). Extracted variables included study design (country, blinding, randomization, analysis population, and follow-up duration), population characteristics (sample size, age, sex, HF phenotype, LVEF, New York Heart Association (NYHA) class, diabetes prevalence, N-terminal pro b-type natriuretic peptide (NT-proBNP), and estimated glomerular filtration rate (eGFR)), intervention details (drug, dose, and comparator), binary outcomes (events and denominators per arm for all-cause mortality (ACM), HFH, CV death, the composite of CV death and HFH, and serious adverse events (SAEs)), time-to-event data (hazard ratios (HRs) with 95% confidence intervals (CIs)), continuous outcomes (between-group mean differences (MDs) with 95% CI for Kansas City Cardiomyopathy Questionnaire (KCCQ) total symptom score, KCCQ overall summary score, six-minute walk distance (6MWD), NT-proBNP, eGFR change, body weight change, and systolic blood pressure (SBP) change), and safety outcomes (diabetic ketoacidosis (DKA), acute kidney injury (AKI), urinary tract infection (UTI), hypotension, and genital infection). When studies reported medians with interquartile ranges rather than means with standard deviations, the Wan estimator was applied [13]. All imputations were documented per study and outcome (imputation formulae in Appendix D).

Risk-of-Bias Assessment

RoB was assessed independently by both reviewers (VMF and VAM) for all 114 included studies using the Cochrane Risk of Bias 2.0 (RoB 2.0) tool for randomized trials [14], evaluating five domains: bias arising from the randomization process (D1), bias due to deviations from intended interventions (D2), bias due to missing outcome data (D3), bias in the measurement of the outcome (D4), and bias in the selection of the reported result (D5). Each domain and the overall judgment were rated as low ROB, some concerns, or high ROB. Disagreements were resolved by discussion. Formal inter-rater reliability statistics (Cohen's kappa, intraclass correlation coefficient (ICC)) were not prospectively recorded.

Statistical Analysis

For binary outcomes, risk ratios (RRs) were calculated using the Mantel-Haenszel method. For studies with zero events in one arm, a continuity correction of 0.5 was added to all cells of the corresponding 2 x 2 table; studies with zero events in both arms were excluded. As a sensitivity analysis, hazard ratios were pooled from studies reporting time-to-event data using generic inverse-variance methods. For continuous outcomes, mean differences were calculated using the inverse-variance method. All analyses used random-effects models with restricted maximum likelihood (REML) estimation and Hartung-Knapp-Sidik-Jonkman (HKSJ) confidence intervals [15]. Heterogeneity was assessed using the Cochran Q test, I^2^ statistic, tau^2^, and 95% prediction intervals (when three or more studies were available) [16]. Heterogeneity was classified as low (I^2^ less than 25%), moderate (25-50%), substantial (50-75%), or high (greater than 75%).

Pre-specified subgroup analyses were performed by HF phenotype (HFrEF, HFpEF, HFmrEF), drug class (empagliflozin, dapagliflozin, canagliflozin, sotagliflozin, other), blinding status (double-blind, open-label, single-blind), follow-up duration (less than 12 months, 12 months or more), sample size (less than 100, 100-999, 1,000 or more), ROB (low, some concerns, high), and publication era (pre-2020, 2020-2021, 2022 or later). Between-subgroup differences were tested using a chi-squared test for interaction. Thirteen filter-based sensitivity analyses and 5 alternative statistical models (fixed-effect Mantel-Haenszel RR, random-effects odds ratio [OR], fixed-effect OR, Peto OR, and Paule-Mandel tau2 estimator) were examined. Leave-one-out analysis and influence diagnostics were performed.

Funnel plot asymmetry was assessed visually for outcomes with three or more studies. Egger's regression test was performed when 10 or more studies were available. When significant asymmetry was detected, the Duval and Twomey trim-and-fill method was applied [17]. Cumulative meta-analysis ordered by publication year was conducted for binary outcomes. Trial sequential analysis (TSA) was performed for primary outcomes at relative risk reduction (RRR) thresholds of 15% and 20%, with type I error of 5% and power of 80%, using O'Brien-Fleming alpha-spending boundaries [18]. Certainty of evidence was assessed using the GRADE approach [19] for each outcome, considering downgrades for ROB, inconsistency, indirectness, imprecision, and publication bias. The number needed to treat (NNT) was calculated for binary outcomes as the inverse of the absolute risk reduction, using the pooled control event rate (CER) as the baseline risk over the sample-size-weighted mean follow-up duration of contributing trials. All analyses were performed in R version 4.4 (R Core Team, 2024) using the meta (version 8.0) [20], metafor (version 4.6) [21], dmetar, and RTSA (version 0.2) packages.

Handling of Multiple Publications per Trial

When two reports presented overlapping populations from the same trial (e.g., a primary HF subgroup paper and a companion sub-analysis), the most recent and most population-comprehensive paper was used for the primary outcome contribution; companion papers were used only for outcomes the primary did not report or were retained in the trial-cluster table without contributing duplicate weight to the meta-analysis pool. All primary publications appear in the reference list and are cited inline in figure labels and per-study table rows.

Findings

Study Selection

**Figure 1:**
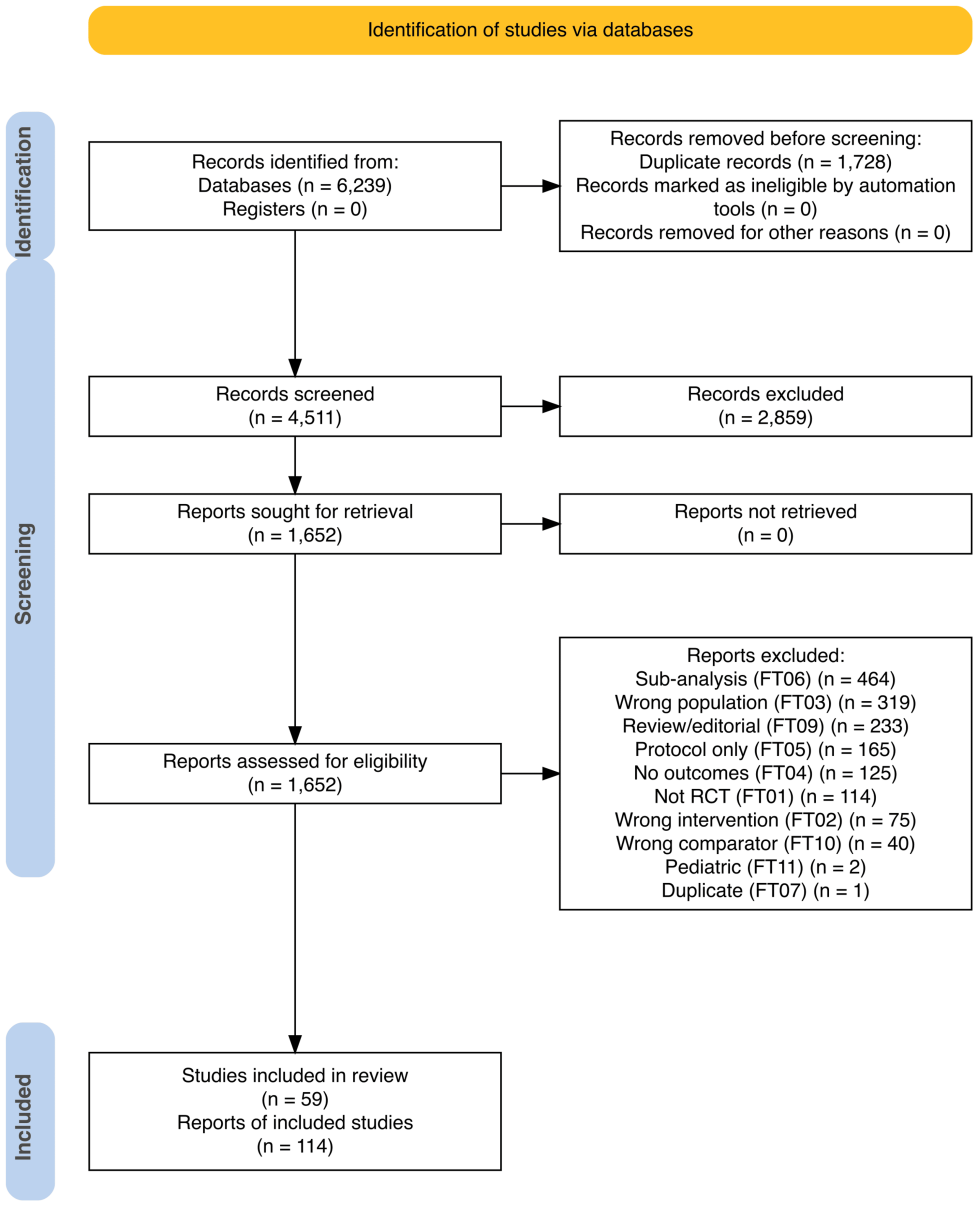
PRISMA 2020 flow diagram of study selection Flow of records through identification, screening, eligibility assessment, and inclusion in the systematic review and meta-analysis. Generated with the Preferred Reporting Items for Systematic reviews and Meta-Analyses (PRISMA) 2020 R package [22].

The search identified 6,239 records across four databases: PubMed (n = 2,243), Cochrane CENTRAL (n = 2,064), ClinicalTrials.gov (n = 1,637), and WHO ICTRP (n = 295). After removing 1,728 duplicates, 4,511 unique records were screened by title and abstract, of which 2,859 were excluded. Full-text assessment of 1,652 reports led to the exclusion of 1,538 reports, with 114 studies meeting inclusion criteria. Of these, 55 were excluded from quantitative synthesis, leaving 59 RCTs in the meta-analysis (Figure 1). The characteristics of the 59 included trials are presented in Table 3, and the 55 excluded records are detailed in Table 4.

**Table 3:** Characteristics of the included studies Per-trial characteristics of the 59 included randomized controlled trials, sorted by reference number. The Ref column links each row to the numbered reference in the reference list.

Ref	record_id	Study	trial_name	publication_year	country	study_type	blinding	hf_type	n_randomized	n_int	n_ctrl	drug_name	drug_dose_mg	comparator	follow_up_weeks	age_mean	female_pct	lvef_mean	diabetes_pct	overall_judgment
[4]	CO-0432	McMurray_2019	DAPA-HF	2019	Multinational (20 countries)	RCT	Double-blind	HFrEF	4744	2373	2371	Dapagliflozin	10	Placebo	79	66.35	23.8	31.05	41.8	Low
[5]	CO-1013	Packer 2021	EMPEROR-Reduced	2021	Multinational (20 countries)	RCT	Double-blind	HFrEF	3730	1863	1867	Empagliflozin	10	Placebo	69.6	NA	NA	NA	NA	Low
[6]	CO-0441	Solomon_2022	DELIVER	2022	20 countries (multinational)	RCT	Double-blind	HFmrEF/HFpEF (LVEF >40%)	6263	3131	3132	dapagliflozin	10	placebo	120	71.65	43.9	54.15	44.8	Low
[7]	CO-0064	Anker 2021	EMPEROR-Preserved	2021	Multinational (23 countries)	RCT	Double-blind	HFpEF	5988	2997	2991	Empagliflozin	10	Placebo	113.7	71.9	44.6	54.3	49.1	Low
[12]	CO-0450	Bhatt_2021	SOLOIST-WHF	2021	Multinational (32 countries)	RCT	Double-blind	Mixed (HFrEF and HFpEF; 79.1% LVEF <50%)	1222	608	614	Sotagliflozin	200 (up to 400)	Placebo	39.1	69.5	33.7	NA	100	Some concerns
[23]	CO-0247	Lin 2024	NA	2024	China	RCT	Double-blind	HFrEF	200	100	100	Dapagliflozin	10	Placebo	104	62.7	33	32.85	41	Some concerns
[24]	CO-0017	Marton 2024	DAPA-Shuttle1	2024	Singapore	phase 4	Double-blind	HFrEF	40	15	14	dapagliflozin	10	placebo	4	59	10.3	31	48.3	Some concerns
[25]	CO-0171	McMurray 2024	DETERMINE	2024	International (multinational)	RCT (2 parallel trials)	Double-blind	HFrEF and HFpEF (two separate cohorts)	817	409	408	dapagliflozin	10	placebo	16	NA	NA	NA	NA	Some concerns
[26]	CO-1252	Mocan 2025	NA	2025	Romania	RCT	Open-label	AHF (any EF)	100	50	50	dapagliflozin	10	structured IV furosemide alone	4	63.63	18.4	26.03	20.4	High
[27]	CO-0025	Mordi 2020	RECEDE-CHF	2020	United Kingdom	crossover RCT	Double-blind	HFrEF	23	23	23	empagliflozin	25	placebo	14	69.8	26.1	NA	100	Low
[28]	CO-0789	Nassif 2019	DEFINE-HF	2019	United States	RCT	Double-blind	HFrEF	263	131	132	dapagliflozin	10	placebo	13	61.3	27	26	62	Some concerns
[29]	CO-0604	Nassif 2021	CHIEF-HF	2022	USA	RCT	Double-blind	HFrEF+HFpEF	476	222	226	canagliflozin	100	placebo	12	63.4	44.9	NA	27.9	Some concerns
[30]	CO-0257	Nassif_2020	EMBRACE-HF	2020	United States	RCT	Double-blind	HFrEF+HFpEF	65	33	32	empagliflozin	10	placebo	13	66	37	44	52	Low
[31]	CO-0408	Nassif_2021	PRESERVED-HF	2021	United States	RCT	Double-blind	HFpEF	324	162	162	dapagliflozin	10	placebo	13	70	57	60	56	Low
[32]	CO-1056	NCT03448419 (EMPERIAL-Reduced)	EMPERIAL-Reduced	2018	USA; Australia; Canada; Germany; Greece; Italy; Norway; Poland; Portugal; Spain; Sweden	phase 3	Double-blind	HFrEF	312	156	156	empagliflozin	10	placebo	12	69	25.6	30	59.9	Low
[32]	CO-1118	Abraham 2021	EMPERIAL	2021	USA; Australia; Canada; Germany; Greece; Italy; Norway; Poland; Portugal; Spain; Sweden	phase 3	Double-blind	HFpEF	315	157	158	empagliflozin	10	placebo	12	73.5	43.2	53.1	51.1	Low
[33]	CO-0529	Omar_2022	Empire HF Biomarker	2022	Denmark	substudy of RCT (Empire HF)	Double-blind	HFrEF	190	95	95	empagliflozin	10	placebo	12	64	15	29	12	Some concerns
[33]	CO-0839	Jensen 2020	Empire HF	2020	Denmark	RCT	Double-blind	HFrEF	190	95	95	empagliflozin	10	placebo	12	64	15	29	18	Some concerns
[34]	CO-0974	Ovchinnikov 2021	Ovchinnikov 2021	2021	Russia	RCT	Open-label	HFpEF	60	30	30	empagliflozin	10	Standard care	24	66.5	61.7	60	100	High
[35]	CO-0389	Ovchinnikov_2025	10.1186/s12933-025-02756-y	2025	Russia	RCT	Open-label	HFpEF	70	35	35	empagliflozin	10	standard care	24	67.1	63	61	100	High
[36]	CO-0697	Palau 2022	DAPA-VO2	2022	Spain	RCT	Double-blind	HFrEF	90	45	45	dapagliflozin	10	placebo	12	68.6	23.3	33.8	32.2	Low
[37]	CO-0062	Pastore 2024	DAPA ECHO	2024	Italy	RCT	Open-label	HFrEF and HFmrEF	88	44	44	Dapagliflozin	10	Optimal medical therapy (OMT) without SGLT2i	26	68	17	37	0	High
[38]	CO-0454	Rau_2021	EudraCT 2016-000172-19	2021	Germany	RCT	Double-blind	T2D with ASCVD (43% chronic HF)	44	20	22	empagliflozin	10	placebo	12	62	19	NA	100	Some concerns
[39]	CO-1139	Reis 2022	NA	2022	Portugal	RCT	Open-label	HFrEF	40	20	20	Dapagliflozin	10	Optimal medical therapy	26	60.9	17.5	34.1	0	High
[40]	CO-0038	Santos-Gallego 2021	EMPA-TROPISM	2021	United States	RCT	Double-blind	HFrEF	84	42	42	empagliflozin	10	placebo	24	NA	NA	36	0	Low
[41]	CO-0110	Schulze 2022	EMPAG-HF	2022	Germany	RCT	Double-blind	Mixed (HFrEF + HFpEF)	60	30	29	Empagliflozin	25	Placebo	4.3	74.7	38	44.5	39	Low
[42]	CO-0600	Shirakabe_2020	Shirakabe 2020	2020	Japan	prospective RCT	Open-label	compensated HF (mixed EF)	60	28	30	empagliflozin	10-25	no treatment (standard care)	26	74	17.2	55	100	High
[43]	PM-0539	Shoshina 2026	NA	2026	Russia	RCT	Open-label	HFpEF	50	25	25	dapagliflozin	10	Standard care	26	63.75	42	NA	6	High
[44]	CO-1283	Tamaki 2021	Tamaki 2021	2021	Japan	RCT	Open-label	mixed (HFrEF 49%/HFmrEF 14%/HFpEF 37%)	62	30	29	empagliflozin	10	conventional glucose-lowering therapy	1	NA	39	NA	100	Some concerns
[45]	CO-1678	Tanaka 2020	CANDLE	2020	Japan	RCT	Open-label	mixed (71% HFpEF; 29% HFrEF)	245	122	123	canagliflozin	100	glimepiride	24	68.6	25	57.6	100	Some concerns
[46]	CO-1542	Thiele 2022	NA	2022	Germany	RCT	Double-blind	mixed	19	10	9	empagliflozin	10	placebo	4.3	72.1	52.6	36	26.3	Some concerns
[47]	CO-0225	Ueda 2021	CANONICAL	2021	Japan	RCT	Open-label	HFpEF	82	42	40	Canagliflozin	100	Standard diabetic therapy	24	75.7	32.9	61.5	100	High
[48]	CO-0028	Voors 2022	EMPULSE	2022	Multinational (15 countries)	RCT	Double-blind	Both (HFrEF and HFpEF; acute de novo and decompensated chronic)	530	265	265	Empagliflozin	10	Placebo	13	70.3	33.8	NA	45.3	Low
[49]	WH-0025	Wu 2022	10.3760/cma.j.cn112148-20220120-00059	2021	China	RCT	Open-label	HFmrEF	112	57	55	empagliflozin	10	conventional therapy	26	69	25	NA	NA	High
[50]	CO-0642	Xie 2024	DAHOS	2024	China	RCT	Open-label	HFrEF	120	60	60	dapagliflozin	10	optimized HF therapy	12	62.1	27.1	33	16.82	High
[51]	CO-1777	Zhou 2024	NA	2024	China	RCT	Single-blind	HFrEF/HFmrEF	98	50	48	dapagliflozin	10	standard care	52	66.63	40.8	48.67	56.12	Some concerns
[52]	CO-0121	Abdullaev 2024	NA	2024	Russia	RCT	Open-label	Mixed (NYHA III-IV)	119	60	59	Dapagliflozin/Empagliflozin	10	Standard diuretic therapy (loop diuretics only)	4.3	71.9	45.4	37.4	100	High
[53]	CO-0383	Abedi_2024	Abedi 2024	2024	Iran	RCT	Double-blind	HFrEF and HFmrEF	80	35	37	Empagliflozin	10	Placebo	24	60.27	22.2	24.73	29.2	Some concerns
[54]	CO-1126	Akasaka 2022	EXCEED	2022	Japan	RCT	Open-label	HFpEF	73	36	32	Ipragliflozin	50	Conventional treatment	24	71.1	39.7	60.7	100	High
[55]	CO-0628	Asif 2024	NA	2024	Pakistan	RCT	Double-blind	HFmrEF	480	240	240	dapagliflozin	10	placebo	4	52.85	44	NA	69.38	High
[56]	CO-0029	Ejiri 2020	MUSCAT-HF	2020	Japan	RCT	Open-label	HFpEF	169	86	83	luseogliflozin	2.5	voglibose	24	73.1	37.6	57.5	100	High
[57]	CO-1700	Golubovskaya 2025a	Golubovskaya 2025a	2025	Russia	RCT	Open-label	HFrEF/HFmrEF/HFpEF	92	46	46	empagliflozin	10	standard care	26	NA	31.5	NA	43.5	High
[58]	CO-0181	Kolwelter 2021a	NCT03128528	2021	Germany	Parallel RCT	Double-blind	Mixed (HFrEF 61% + HFmrEF)	75	48	26	Empagliflozin	10	Placebo	13	66	15	39	23	Some concerns
[59]	CO-0380	Bhushan_2023	Remo Safe-AHF	2023	India	RCT	Open-label	HFrEF	35	17	18	Remogliflozin	NA	Conventional therapy	12	NA	NA	29.87	100	High
[60]	CO-0040	Borlaug 2023	CAMEO-DAPA	2023	United States	RCT	Double-blind	HFpEF	38	21	17	dapagliflozin	10	placebo	25	67	66	62	18	Low
[61]	CO-0106	Charaya 2023	NA	2023	Russia	RCT	Open-label	HFrEF and HFmrEF and HFpEF (ADHF)	200	94	106	Dapagliflozin	10	Standard therapy for ADHF (IV loop diuretics)	4	74	49	47	31	High
[62]	CO-0726	Damman 2020	EMPA-RESPONSE-AHF	2020	Netherlands	RCT	Double-blind	acute HF	80	41	39	empagliflozin	10	placebo	8.6	76	33	36	33	Some concerns
[63]	CO-0951	de Boer 2020	de Boer 2020 (licogliflozin)	2020	21 countries	RCT	Double-blind	HFrEF + HFpEF (NYHA II-IV; mixed LVEF)	125	62	33	licogliflozin	2.5/10/50	placebo	12	NA	28.2	NA	100	Some concerns
[64]	CO-0399	EFFORT	10.1161/CIRCULATIONAHA.124.069144	2020	South Korea	RCT	Double-blind	HFmrEF	128	63	65	ertugliflozin	NA	placebo	52	66.4	39	42	12.5	Low
[65]	CO-1544	Emara 2023	DAPA-RESPONSE-AHF	2023	NA	RCT	Double-blind	Acute HF	87	45	42	dapagliflozin	10	Placebo	4	NA	NA	NA	NA	Some concerns
[66]	CO-0933	Fatima Gilani 2023	NA	2023	Pakistan	RCT	Open-label	AHF (LVEF <40%)	160	80	80	dapagliflozin	10	standard medical therapy (placebo-free)	1	65.13	18.8	NA	51.25	High
[67]	CO-1258	Fatima Gilani 2024	NA	2024	Pakistan	RCT	Open-label	AHF (EF <40%)	150	75	75	dapagliflozin	10	conventional therapy alone	12	63.76	17.3	NA	48	High
[68]	CO-0466	Fu_2023	NA	2023	China	RCT	Double-blind	HFrEF	60	30	30	dapagliflozin	10	placebo	52	70.55	28.3	30.95	100	Some concerns
[69]	CO-0482	Gojaseni 2024	CO-0482	2024	Thailand	RCT	Open-label	acute decompensated HF	32	12	13	dapagliflozin	10	standard of care	4	67.1	51.5	42.1	NA	High
[70]	CO-0161	Griffin 2020	Griffin 2020	2020	USA	Crossover RCT	Double-blind	Mixed (HFrEF 45% + HFpEF)	20	20	20	Empagliflozin	10	Placebo	6	60	25	42.9	100	Some concerns
[71]	CO-1011	Hundertmark 2023	EMPA-VISION	2023	UK	RCT	Double-blind	HFrEF+HFpEF	72	35	37	empagliflozin	10	Placebo	12	68.33	41.7	NA	12.5	Some concerns
[72]	CO-1015	Ilyas 2021	NA	2021	Australia	crossover RCT	Double-blind	HFrEF	19	19	19	dapagliflozin	10	placebo	8	73	26	35	100	Some concerns
[73]	CO-1251	Kawanami 2025	ROAD-ADHF	2025	Japan	RCT	Open-label	HFrEF/HFmrEF	117	56	58	dapagliflozin	10	conventional therapy (loop diuretics alone)	1	73	35	33	NA	High
[74]	CO-0035	Lee 2021	SUGAR-DM-HF	2021	United Kingdom (Scotland)	RCT	Double-blind	HFrEF	105	52	53	empagliflozin	10	placebo	36	68.7	26.7	32.5	78.1	Low

**Table 4:** Characteristics of studies excluded from the quantitative synthesis Per-record details of the 55 studies included in the systematic review but excluded from the meta-analysis, sorted by reference number. The Ref column carries an own reference-list number for the 21 citable excluded reports, or the parent trial's number for the 34 records that duplicate or sub-analyze a trial already among the 59 in the quantitative synthesis.

Ref	Study	Trial	Exclusion reason	Parent
[4]	NCT03036124 2017	DAPA-HF	Duplicate: DAPA-HF registry (CO-0432)	CO-0432
[5]	EUCTR2016-002280-34-DE 2016	EMPEROR-Reduced	Duplicate: EMPEROR-Reduced EUCTR (CO-1013)	CO-1013
[5]	NCT03057977 2017	EMPEROR-Reduced	Duplicate: EMPEROR-Reduced registry (CO-1013)	CO-1013
[5]	Spinar 2020	EMPEROR-Reduced	Sub-analysis: EMPEROR-Reduced companion (CO-1013)	CO-1013
[5]	Spinar 2020	EMPEROR-Reduced	Duplicate: EMPEROR-Reduced summary (CO-1013)	CO-1013
[7]	Packer 2021	EMPEROR-Preserved (WHF)	Sub-analysis: EMPEROR-Preserved WHF subgroup (CO-0064)	CO-0064
[12]	Kosiborod 2023	SOLOIST-WHF	Sub-analysis: SOLOIST-WHF KCCQ (CO-0450)	CO-0450
[12]	NCT03521934 2018	SOLOIST-WHF	Duplicate: SOLOIST-WHF registry (CO-0450)	CO-0450
[25]	NCT03877237 2019	DETERMINE-reduced	Registry only: DETERMINE duplicate (CO-0171)	CO-0171
[27]	Mordi 2021	RECEDE-CHF	Duplicate: RECEDE-CHF crossover sub-study (CO-0025 is primary)	CO-0025
[29]	NCT04252287 2020	CHIEF-HF	Duplicate: CHIEF-HF registry (CO-0604)	CO-0604
[29]	Mohebi 2024	CHIEF-HF	Sub-analysis: CHIEF-HF health status (CO-0604)	CO-0604
[31]	NCT03030235 2017	PRESERVED-HF	Duplicate: PRESERVED-HF registry (CO-0408)	CO-0408
[33]	Omar 2021	Omar 2021 (post hoc)	Sub-analysis of Empire HF (CO-0529), conference abstract	CO-0529
[36]	Lorenzo 2023a	DAPA-VO2	Sub-analysis: DAPA-VO2 iron kinetics (CO-0697)	CO-0697
[36]	Lorenzo 2023b	DAPA-VO2	Sub-analysis: DAPA-VO2 hemoglobin (CO-0697)	CO-0697
[37]	Pastore 2024b	DAPA ECHO	Duplicate: DAPA ECHO conference (CO-0062)	CO-0062
[48]	NCT04157751 2019	EMPULSE	Duplicate: EMPULSE registry (CO-0028)	CO-0028
[49]	Wu 2022	—	Duplicate of WH-0025 (same PMID 35856224, same DOI) - FT07	WH-0025
[50]	Xie 2023	DAHOS	Protocol only: DAHOS protocol (CO-0642 has results)	CO-0642
[55]	Ali 2024	—	Duplicate: likely same dataset as CO-0628	CO-0628
[56]	Ejiri 2019	MUSCAT-HF	Duplicate: MUSCAT-HF earlier report (CO-0029)	CO-0029
[57]	Golubovskaya 2023	Golubovskaya 2023	Duplicate: Golubovskaya early report (CO-1700)	CO-1700
[57]	Golubovskaya 2025b	Golubovskaya 2025b	Duplicate: Golubovskaya 12-month (CO-1700 is 6-month primary)	CO-1700
[58]	Bosch 2023	ELSI (post hoc)	Sub-analysis: NCT03128528 post-hoc (CO-0181)	CO-0181
[58]	Kolwelter 2021b	NCT03128528	Sub-analysis: NCT03128528 conference (CO-0181)	CO-0181
[58]	Kolwelter_2023	ELSI	Duplicate: ELSI earlier conference (CO-0181 is primary)	CO-0181
[58]	Kolwelter 2021c	NCT03128528	Sub-analysis: NCT03128528 conference (CO-0181)	CO-0181
[58]	Kolwelter 2021d	NCT03128528	Sub-analysis: NCT03128528 conference (CO-0181)	CO-0181
[60]	Naser_2024	CAMEO-DAPA (secondary)	Sub-analysis: CAMEO-DAPA body composition (CO-0040)	CO-0040
[62]	Boorsma 2021	EMPA-RESPONSE-AHF	Sub-analysis: EMPA-RESPONSE-AHF biomarker (CO-0726)	CO-0726
[73]	Kawanami 2024	ROAD-ADHF	Duplicate: ROAD-ADHF conference (CO-1251)	CO-1251
[75]	Charaya 2022	—	Pilot study (n=102) of same NCT04778787 as CO-0106 (n=200) (FT06)	—
[76]	Charaya 2023	—	FT06: Secondary analysis of CO-0106 trial (NCT04778787, Charaya/Sechenov)	—
[77]	EASTER-HF	10.1016/j.ihj.2024.06.009	Not an RCT (retrospective observational study) — FT01	—
[78]	EUCTR2021-005446-15-NL	DAPA-severe-CKD	Registry only: no results (CKD population, not HF)	—
[79]	Jones 2020	—	HLC 2020 conference abstract, same trial as CO-1015 Ilyas 2021 (FT06)	—
[80]	Kumar 2024	—	FT04: PICO mismatch (T2DM+CVD, not HF) + suspected data fabrication	—
[81]	Maddukuri_2025	—	Full-text not available/locatable (FT05)	—
[82]	Montomoli 2024	DAPA-DP	Protocol only: no results	—
[83]	Murakami 2017	Murakami 2017	Full-text not available/locatable (FT05), abstract only	—
[84]	NCT04249778	DAPA-Discharge	Registry only: no results	—
[85]	NCT04869124	DAPA-VOLVO	Registry only: no results	—
[86]	NCT05152940 (ERTU-SODIUM)	ERTU-SODIUM	Registry only: no results	—
[87]	NCT06012279 (CODA-HFrEF)	CODA-HFrEF	Registry only: no results	—
[88]	Polat_2025	CO-0368	Non-RCT (observational with propensity score matching)	—
[89]	Qin 2023	Qin 2023	ADA 2023 late-breaking abstract, n=6 (FT09)	—
[90]	Singh 2019	REFORM	Scottish Medical Journal conference abstract book (FT09)	—
[91]	Tada_2024	—	ESC 2024 congress abstract + secondary analysis of RCT (FT06)	—
[92]	Tamaki 2019	Tamaki 2019	Sub-analysis: Tamaki UA sub-analysis	—
[93]	Tamaki_2020	Tamaki 2020	Sub-analysis: Tamaki TTKG sub-analysis	—
[94]	You 2025	—	HLC 2025 conference abstract only (FT09)	—
[95]	Zaoui 2022	10.1016/j.ancard.2022.08.007	Non-RCT: non-randomized study	—
[96]	Murakami 2016	Murakami 2016	Duplicate: Murakami 2016 earlier version (CO-1747)	CO-1747
[97]	Singh 2018	—	Duplicate: Singh 2018 = REFORM pilot (CO-0800 is primary)	CO-0800

Study Characteristics

The 59 included RCTs randomized 29,692 participants (14,848 to SGLT2 inhibitors, 14,805 to control) across 30 countries, published between 2018 and 2026 [4-7,12,23-74] (Table 3). These 59 studies are reported across 58 primary references; Abraham et al. [32] report both EMPERIAL-Preserved and EMPERIAL-Reduced. Empagliflozin was investigated in 25 trials (42%), dapagliflozin in 24 (41%), canagliflozin in three, sotagliflozin (dual SGLT1/SGLT2) in one, and other SGLT2 inhibitors (ipragliflozin, luseogliflozin, and tofogliflozin) in six. With respect to HF phenotype, 17 trials (29%) enrolled exclusively HFrEF patients, 10 (17%) HFpEF, three (5%) HFmrEF, and 29 (49%) enrolled mixed populations or did not specify HF phenotype. Thirty-six trials (61%) were double-blind, 22 (37%) were open-label, and one was single-blind. The median follow-up was 13 weeks (range 1-120 weeks). The median of mean age across trials was 68 years, with a median female proportion of 33%. The median of mean LVEF was 37%.

Excluded Studies

Of the 114 studies meeting inclusion criteria, 55 were excluded from quantitative synthesis (Table 4): 21 carry their own number in the reference list [75-95], and 34 are duplicate reports, registry entries, or sub-analyses of a trial already among the 59 and inherit that parent trial's reference number. The 21 citable excluded reports include observational or non-randomized reports, conference abstracts with their own publication record, protocol papers with their own digital object identifier, registry entries for trials without results yet published (referenced by their ClinicalTrials.gov or EUCTR URL), and one record (Kumar, 2024) retained for narrative review because of the integrity concern described next. Kumar (2024) (n = 250, empagliflozin versus placebo) reported hazard ratios for CV outcomes numerically identical to those of the EMPA-REG OUTCOME trial (n = 7,020) - a statistical near-impossibility for an independent 250-participant study; this record also met the FT04 exclusion criterion (population with type 2 diabetes mellitus and CV risk, not HF) and is cited as a Table 4 entry for transparency.

Risk of Bias

**Figure 2:**
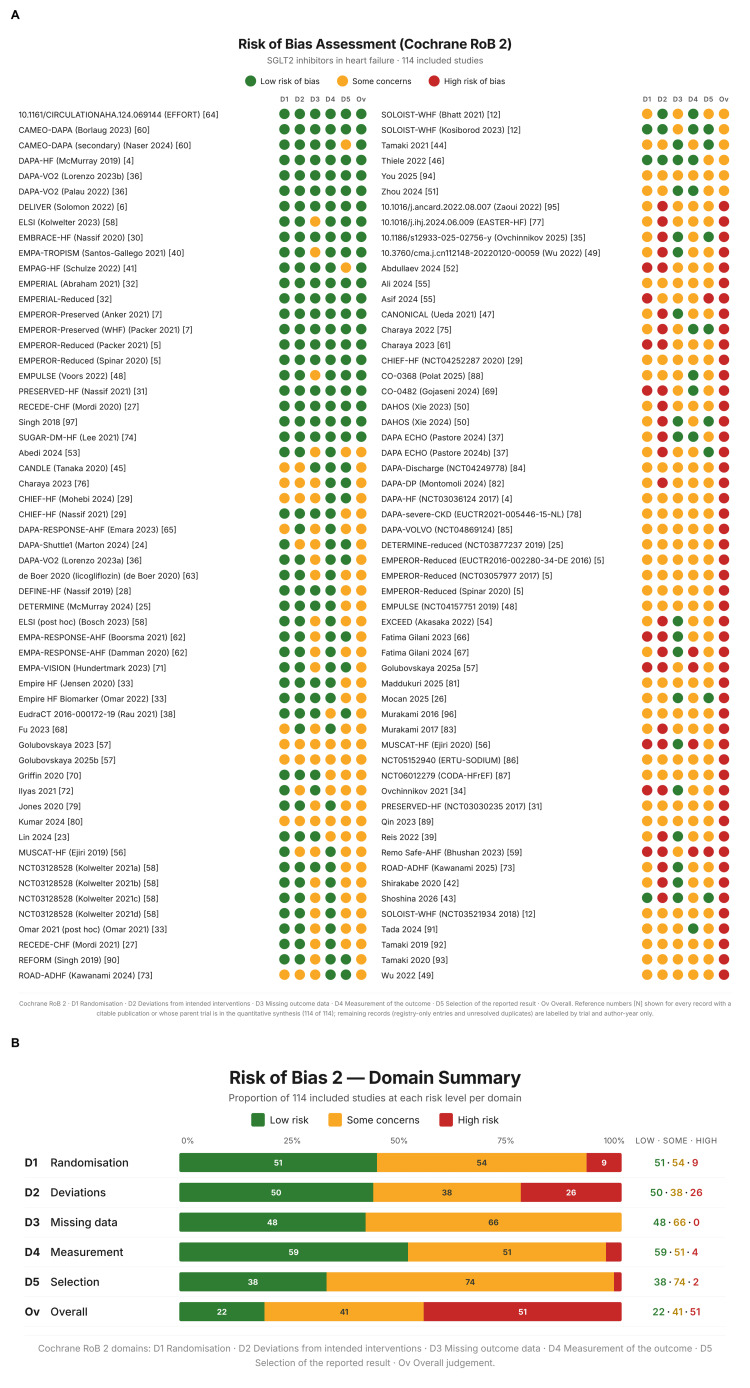
Risk of bias 2.0 assessment: traffic light grid (Panel A) and domain summary (Panel B) Panel A: Per-study risk-of-bias judgments across five Cochrane RoB 2.0 domains plus overall judgment for all 114 included studies, sorted as Low → Some concerns → High, then alphabetically within group. Reference numbers in [N] format are shown for every record: the 59 trials in the quantitative synthesis plus the 21 citable excluded reports (observational reports, conference abstracts, and protocol papers with their own digital object identifier) carry their own number, while records that duplicate or sub-reference a trial already in the quantitative synthesis carry that parent trial's reference number. Panel B: Domain-level summary across all 114 included trials.

Among the 114 included RCTs, 22 (19%) were assessed as low ROB overall, 41 (36%) as some concerns, and 51 (45%) as high ROB (Figure 2). The most common sources of concern were domain D2 (deviations from intended interventions), reflecting the proportion of open-label trials, and domain D5 (selection of reported results), particularly in smaller single-center studies lacking prospective registration or statistical analysis plans. Within the 59 trials contributing to the quantitative synthesis, the distribution was more favorable (16 (27%) = low, 22 (37%) = some concerns, 21 (36%) = high risk), as the larger registered multinational trials were preferentially poolable.

Primary Outcomes

**Figure 3:**
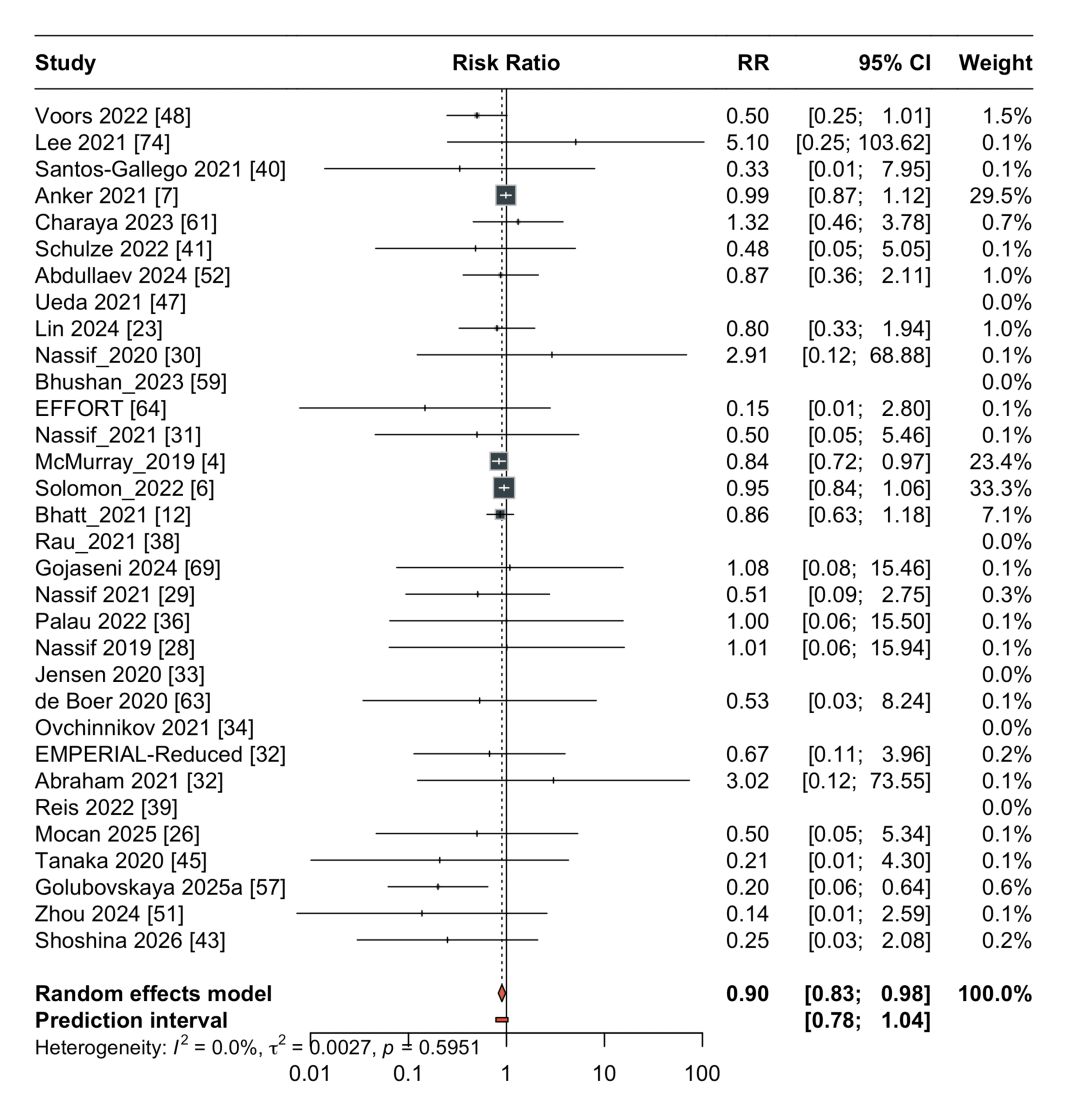
Forest plot: all-cause mortality Random-effects pooled risk ratio for all-cause mortality across 26 randomized controlled trials with at least one event. Studies sorted by reference number; reference numbers in [N] format inline with each study label.

All-cause mortality data were available in 32 trials. Six trials had zero events in both arms and were excluded from the pooled estimate; seven additional trials had zero events in one arm (continuity correction of 0.5 applied). Thus, 26 trials with at least one event in at least one arm contributed to the pooled analysis, of which only 19 had events in both arms. SGLT2 inhibitors were associated with a reduction in ACM (RR 0.90, 95% CI 0.83-0.98; p = 0.016), with an I^2^ of 0% (tau^2^ = 0.003; Figure 3). The apparent absence of heterogeneity should be interpreted cautiously: when many contributing trials are small with sparse events, the I^2^ statistic has low power to detect between-study variance. The 95% prediction interval was 0.78 to 1.04, indicating that the treatment effect in a future trial could plausibly include no benefit. In a sensitivity analysis using hazard ratios from only four trials reporting time-to-event data, the point estimate was in the same direction but did not reach statistical significance (HR 0.92, 95% CI 0.80-1.06; p = 0.154). The NNT to prevent one death was 78 (CER 12.8% over a sample-size-weighted mean follow-up of 85.7 weeks). The certainty of evidence was rated as low, downgraded for ROB (31% of contributing studies had a high RoB) and publication bias (Egger's test p = 0.028). Trim-and-fill analysis imputed six missing studies and yielded an adjusted RR of 0.92 (95% CI 0.84-1.00; p = 0.054), with the confidence interval crossing 1.0.

**Figure 4:**
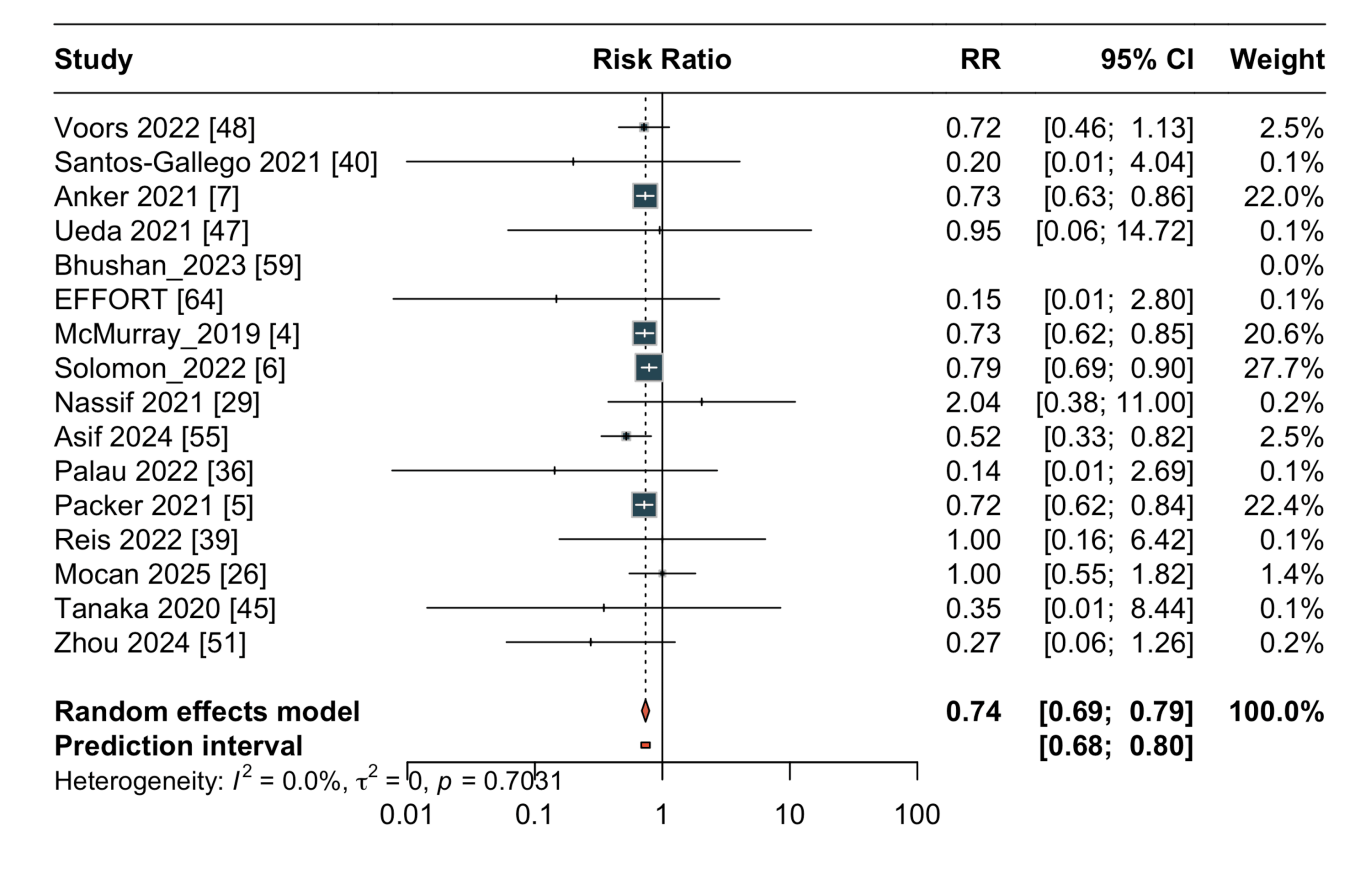
Forest plot: heart failure hospitalization Random-effects pooled risk ratio for heart failure hospitalization across 15 randomized controlled trials. Reference numbers in [N] format inline with each study label.

Fifteen trials reported HFH events. SGLT2 inhibitors significantly reduced HFH (RR 0.74, 95% CI 0.69-0.79; p < 0.001), with no heterogeneity (I2 = 0%; Figure 4). The 95% prediction interval was 0.68 to 0.80. The HR-based sensitivity analysis confirmed this finding (HR 0.72, 95% CI 0.66-0.78; p = 0.001; 4 trials). The NNT was 29 (CER 13.5% over a sample-size-weighted mean follow-up of 91.1 weeks). The certainty of evidence was rated as moderate, downgraded one level for risk of bias (31% high RoB).

Secondary Outcomes

Seven trials contributed to the CV death analysis. SGLT2 inhibitors significantly reduced CV death (RR 0.86, 95% CI 0.76-0.98; p = 0.031; I^2^ = 18%; moderate certainty). The HR-based analysis (four trials) was consistent (HR 0.87, 95% CI 0.80-0.94; p = 0.010). The NNT was 81. The composite endpoint of CV death and HFH was significantly reduced (RR 0.80, 95% CI 0.75-0.85; p < 0.001; eight trials; I2 = 0%; high certainty); the HR-based analysis (eight trials) was concordant (HR 0.77, 95% CI 0.73-0.81; p < 0.001), and the NNT was 24.

Twenty trials reported SAEs. SGLT2 inhibitors were associated with fewer SAEs (RR 0.94, 95% CI 0.90-0.99; p = 0.017; I2 = 36%; high certainty); the NNT was 42. The KCCQ total symptom score was reported in nine trials, with a significant improvement favoring SGLT2 inhibitors (MD 2.6 points, 95% CI 1.2-4.0; p = 0.002; I^2^ = 50%; high certainty). The KCCQ overall summary score (three trials) showed a non-significant trend (MD 2.8 points, 95% CI -1.9 to 7.5; p = 0.127; moderate certainty). 6MWD showed no significant improvement (MD 3.9 meters, 95% CI -6.5 to 14.4; p = 0.406; eight trials; I^2^ = 68%; low certainty). NT-proBNP change was not significantly different (MD -30.0 picograms per milliliter, 95% CI -89.0 to 29.0; p = 0.269; eight trials; I2 = 66%; very low certainty); two studies reporting geometric mean ratios were excluded to avoid scale mixing. Body weight was reduced (MD -1.3 kilograms, 95% CI -2.5 to -0.01; p = 0.048; nine trials; I2 = 84%; moderate certainty). eGFR change (three trials) and SBP change (eight trials) were not significantly different.

Safety outcomes

Genital infections were significantly more common with SGLT2 inhibitors (RR 3.75, 95% CI 1.72-8.19; p = 0.007; six trials; I^2^ = 0%). No significant increase was observed for DKA (RR 1.47, 95% CI 0.10-21.8; p = 0.679; four trials), AKI (RR 0.89, 95% CI 0.63-1.26; p = 0.470; 13 trials), UTI (RR 1.01, 95% CI 0.54-1.88; p = 0.981; 13 trials), or hypotension (RR 0.99, 95% CI 0.69-1.43; p = 0.955; eight trials). Pooled relative risks for all specifically reported adverse events are presented in Table 5.

**Table 5:** Safety outcomes: pooled risk ratios Pooled relative risks for adverse events specifically reported across the SGLT2 inhibitor randomized controlled trial literature.

Outcome	Trials (k)	Risk ratio	95% CI lower	95% CI upper	p-value	I-squared (%)	Direction
Genital infection	6	3.75	1.72	8.19	0.007	0	Increased with SGLT2i
Diabetic ketoacidosis	4	1.47	0.10	21.83	0.679	14	No significant difference
Acute kidney injury	13	0.89	0.63	1.26	0.470	24	No significant difference
Urinary tract infection	13	1.01	0.54	1.88	0.981	0	No significant difference
Hypotension	8	0.99	0.69	1.43	0.955	0	No significant difference

Subgroup Analyses

**Figure 5:**
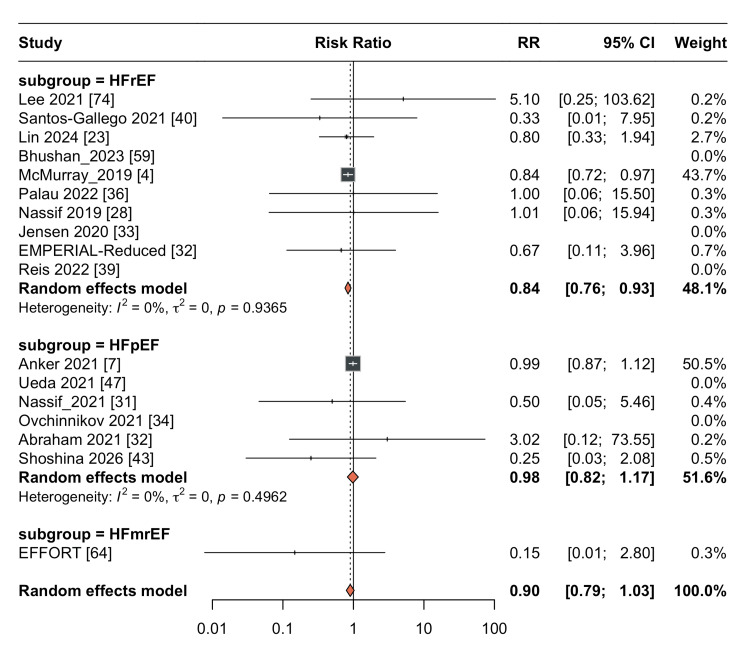
Subgroup forest plot: all-cause mortality by heart failure phenotype Subgroup analysis of all-cause mortality by HF phenotype (HFrEF, HFpEF, and HFmrEF). Reference numbers in [N] format inline with each study label.

For ACM, the test for subgroup differences was significant by HF phenotype (interaction p = 0.039), with a greater benefit in HFrEF (RR 0.84, 95% CI 0.76-0.93; seven trials) than HFpEF (RR 0.98, 95% CI 0.82-1.17; four trials) (Figure 5). Significant interactions were also observed for follow-up duration (p = 0.047), sample size (p = 0.002), and publication era (p = 0.009). No significant interaction was detected for drug class (p = 0.247), blinding status (p = 0.142), or ROB (p = 0.251). For HFH, the benefit was consistent across all subgroups; a significant interaction was observed for blinding (p = 0.018), with a stronger effect in double-blind trials (RR 0.74) than open-label trials (RR 0.97).

Sensitivity Analyses

**Figure 6:**
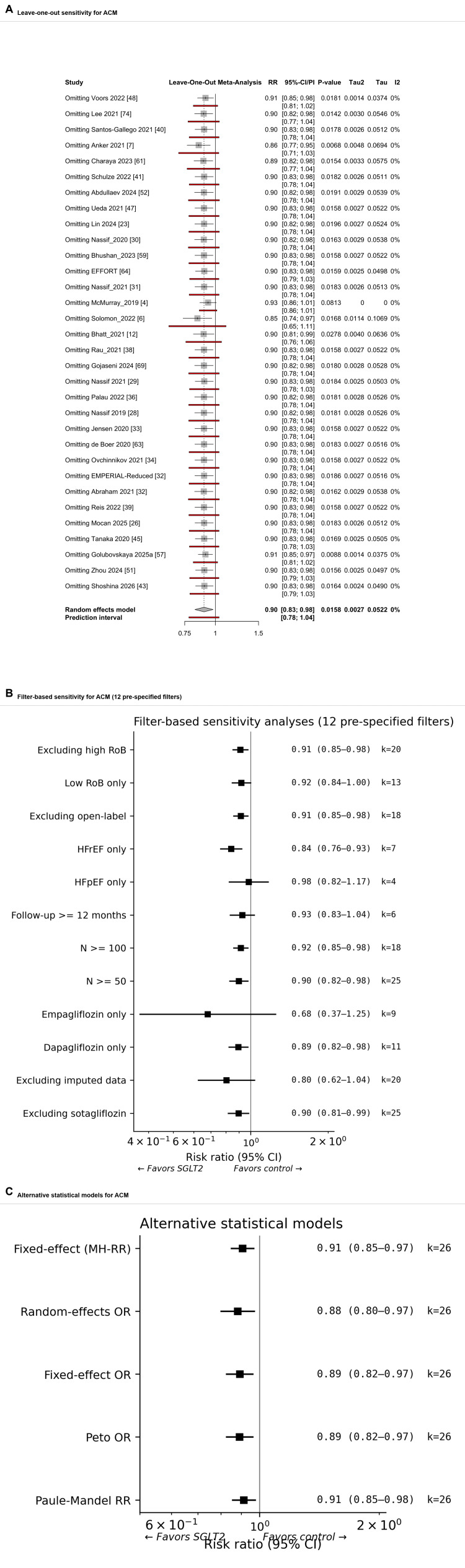
Sensitivity analyses: composite of leave-one-out, filter-based sensitivity, and alternative models Panel A: Leave-one-out sensitivity for all-cause mortality, with each row showing the pooled risk ratio when the corresponding trial is omitted (reference numbers inline with study labels). Panel B. Filter-based sensitivity for all-cause mortality across 12 pre-specified subsets (excluding high risk of bias (RoB), low RoB only, excluding open-label, HFrEF only, HFpEF only, follow-up ≥12 months, sample size thresholds, drug-class restrictions, excluding imputed data, excluding sotagliflozin). Panel C. Alternative statistical models for all-cause mortality (fixed-effect Mantel-Haenszel risk ratio (RR), random-effects odds ratio (OR), fixed-effect OR, Peto OR, Paule-Mandel RR).

The ACM point estimate was directionally consistent across all 13 filter-based sensitivity analyses (range RR 0.68-0.98), although several lost statistical significance (Figure 6). The effect remained significant when excluding high RoB studies (RR 0.91), excluding open-label trials (RR 0.91), and when restricted to dapagliflozin only (RR 0.89). The effect was not significant when restricted to low-RoB studies only (RR 0.92, p = 0.056), HFpEF only (RR 0.98), follow-up of 12 months or longer (RR 0.93), empagliflozin only (RR 0.68), or when excluding studies with imputed data (RR 0.80). Excluding sotagliflozin did not materially change the results. Alternative statistical models yielded consistent results: fixed-effect MH-RR 0.91, random-effects OR 0.88, fixed-effect OR 0.89, Peto OR 0.89, and Paule-Mandel RR 0.91. The Paule-Mandel estimator, which is more robust than REML with sparse binary data, confirmed the direction and magnitude of the primary estimate. The HFH result was consistent across all 13 sensitivity analyses and all five alternative statistical models (all p < 0.001).

Publication Bias and Trial Sequential Analysis

**Figure 7:**
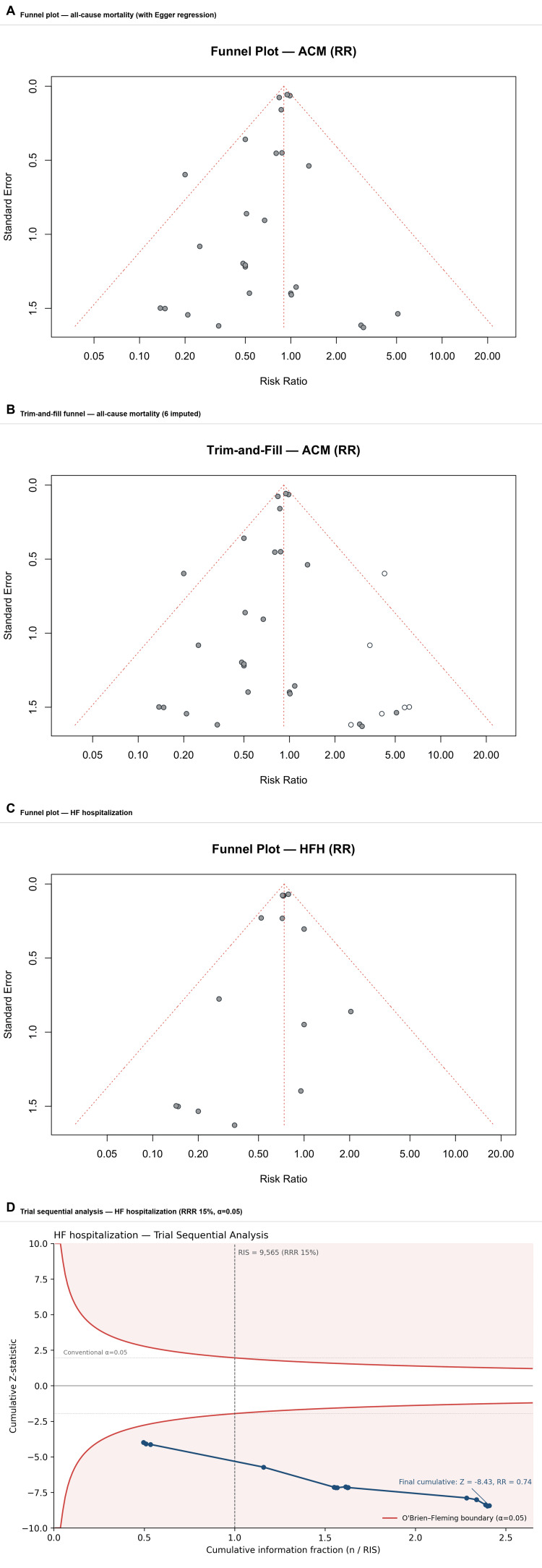
Publication bias and trial sequential analysis: composite Panel A: Funnel plot for all-cause mortality across the contributing trials. Panel B: Trim-and-fill funnel plot for all-cause mortality with six imputed studies. Panel C: Funnel plot for HF hospitalization. Panel D: Trial sequential analysis Z-curve for HF hospitalization at 15% relative risk reduction threshold, with O'Brien-Fleming conservative monitoring boundaries (α = 0.05, β = 0.20); the cumulative Z-statistic crosses the lower boundary well before the required information size, confirming benefit. Funnel plots are aggregate.

Egger's test was significant for ACM (p = 0.028), suggesting possible small-study effects (Figure 7, Panel A). Trim-and-fill analysis imputed six studies and yielded an adjusted RR of 0.92 (95% CI 0.84-1.00; p = 0.054); the adjusted CI crosses 1.0 (Panel B). Egger's test was not significant for HFH (p = 0.189; Panel C) or SAE (p = 0.431). NT-proBNP (k = 8) did not meet the minimum threshold of 10 studies for Egger's test. Cumulative meta-analysis showed that the ACM effect estimate stabilized after 2022 and the HFH estimate was stable throughout the accrual of evidence. Trial sequential analysis for HFH (Panel D), with O'Brien-Fleming conservative monitoring boundaries at a 15% RRR threshold (α = 0.05 two-sided, β=0.20), showed that the cumulative Z-statistic crossed the lower boundary (favoring SGLT2 inhibitors) early in the accrual of evidence and that the required information size of 9,565 participants was exceeded with 23,079 participants contributing across the 15 contributing trials, confirming the robustness of the HFH benefit.

Certainty of Evidence

The GRADE assessment yielded high certainty for the composite of CV death and HFH, SAE, and KCCQ-TSS; moderate certainty for HFH, CV death, KCCQ-OSS, and body weight; low certainty for ACM, 6MWD, and eGFR; and very low certainty for NT-proBNP and SBP (Table 6).

**Table 6:** GRADE summary of findings Outcome-level certainty of evidence per the Grading of Recommendations Assessment, Development and Evaluation (GRADE) framework, with downgrade reasons and pooled effect estimates.

Outcome	k	Certainty	Downgrades	Comments
ACM (RR)	26	Low	Risk of bias (-1); publication bias (-1)	31% high RoB; Egger p = 0.028
HFH (RR)	15	Moderate	Risk of bias (-1)	31% high RoB
CVD (RR)	7	Moderate	Risk of bias (-1)	30% high RoB
Composite (RR)	8	High		
SAE (RR)	20	High		
KCCQ-TSS	9	High		
KCCQ-OSS	3	Moderate	Imprecision (-1)	
6MWD	8	Low	Inconsistency (-1); imprecision (-1)	I² = 68%
NT-proBNP	8	Very low	Risk of bias (-1); inconsistency (-1); imprecision (-1)	38% high RoB; I² = 66%
eGFR	3	Low	Risk of bias (-1); imprecision (-1)	33% high RoB
Weight	9	Moderate	Inconsistency (-1)	I² = 84%
SBP	8	Very low	Risk of bias (-1); inconsistency (-1); imprecision (-1)	38% high RoB; I² = 62%

Interpretation

Summary of Evidence

This systematic review and meta-analysis of 59 RCTs (29,692 participants) demonstrates that SGLT2 inhibitors reduce HFH, CV death, the composite of CV death and HFH, and serious adverse events with moderate-to-high GRADE certainty. The all-cause mortality reduction is of low certainty and sensitive to publication bias. Quality-of-life improvement on the KCCQ total symptom score is supported by high-certainty evidence. Genital infections are the only safety signal demonstrating a significant increase. These findings extend prior pivotal-trial syntheses by incorporating the broader randomized evidence base, including smaller trials with patient-reported outcomes, additional safety signals, and patient subpopulations underrepresented in the landmark trials.

All-Cause Mortality Fragility

The ACM finding merits careful interpretation in light of converging signals of fragility. First, although the pooled estimate was statistically significant, the GRADE certainty was rated as low due to ROB concerns (31% of contributing trials with high RoB) and publication bias (Egger p = 0.028). Second, the trim-and-fill adjusted estimate (RR 0.92, 95% CI 0.84-1.00) crossed 1.0, confirming that the statistical significance of the primary finding is sensitive to potential small-study effects. The funnel plot asymmetry, with numerous small studies clustered in the lower-left quadrant showing implausibly large effects (RR 0.14-0.50), is the classic signature of publication bias or small-study effects rather than genuine treatment benefit. This is further supported by the significant subgroup interaction by sample size (p = 0.002): trials with fewer than 100 participants showed a markedly stronger effect than the large pivotal trials (1,000 or more participants) that drive the overall estimate. Third, the low-RoB-only sensitivity analysis lost significance (RR 0.92, p = 0.056), as did the HFpEF-only restriction (RR 0.98, p = 0.763) and the follow-up-of-12-months-or-longer restriction (RR 0.93, p = 0.142). Fourth, the HR-based sensitivity analysis - which avoids the limitations of continuity correction and directly accounts for time-to-event - showed a non-significant trend in the same direction (HR 0.92, 95% CI 0.80-1.06; p = 0.154) based on only 4 trials. Fifth, the 95% prediction interval (0.78-1.04) crosses 1.0. The ACM benefit is largely driven by HFrEF trials, where the subgroup estimate was significant (RR 0.84) and consistent with the individual trial data from DAPA-HF [4] and EMPEROR-Reduced [5]; no benefit was observed in HFpEF, consistent with DELIVER [6] and EMPEROR-Preserved [7].

Comparison With Prior Meta-Analyses

Prior meta-analyses of the pivotal SGLT2 inhibitor trials [8-9] have consistently shown reductions in HFH and the composite endpoint of comparable magnitude to those reported here. The principal differences in our synthesis are: (1) the inclusion of approximately 50 additional smaller trials providing data on patient-reported outcomes, safety signals, and HF subpopulations; (2) a quantified ACM signal that emerges only when the broader trial set is pooled, but which is concurrently shown to be fragile under sensitivity analysis and publication-bias adjustment; (3) the pre-specified incorporation of sotagliflozin under transparent class-effect assumptions; and (4) the most detailed safety assessment to date for this drug class in HF.

Class Effects and Pharmacological Considerations

No significant heterogeneity was observed between individual SGLT2 inhibitors for either primary outcome (interaction p = 0.247 for ACM, p = 0.424 for HFH). The majority of evidence came from empagliflozin (25 trials) and dapagliflozin (24 trials), with limited data for canagliflozin (three trials) and sotagliflozin (one trial). The empagliflozin-only ACM analysis showed a wider CI and higher heterogeneity (RR 0.68, 95% CI 0.37-1.25; I^2^ = 40%) than the dapagliflozin-only analysis (RR 0.89, 95% CI 0.82-0.98; I^2^ = 0%); this paradox likely reflects the inclusion of several small empagliflozin trials with extreme point estimates and sparse events. While the absence of a significant drug-class interaction supports a class effect, the CIs for the less-studied agents were wide, and firm conclusions about equivalence cannot be drawn. Sotagliflozin is pharmacologically distinct from selective SGLT2 inhibitors (additional intestinal SGLT1 inhibition); a pre-specified sensitivity analysis excluding sotagliflozin did not materially change either primary outcome, supporting the class-effect interpretation while acknowledging the distinction.

Safety considerations

The increased risk of genital infections (RR 3.75) is consistent with the established mechanism (urinary glucose excretion). The absence of significant increases for DKA, AKI, UTI, or hypotension is reassuring, although the wide CIs for DKA reflect the rarity of this event. Clinicians should counsel patients on genital hygiene and the symptoms of urinary tract or genital infection at initiation.

Limitations

Several limitations should be considered. First, approximately 45% of the 114 included trials were rated as high risk of bias overall; this proportion was lower (36%) among the 59 trials in the quantitative synthesis, and among trials contributing to the ACM analysis, the high-RoB proportion was 31%. Sensitivity analyses excluding high-RoB trials did not substantially alter the primary results, but the certainty for ACM was downgraded accordingly. Second, publication bias was detected for ACM, and the trim-and-fill adjusted estimate crossed 1.0. Third, the smaller trials that contributed many of the additional events compared with prior meta-analyses may differ systematically from the large multinational pivotal trials in terms of patient populations, care settings, and outcome ascertainment. Fourth, continuous outcomes were limited by sparse reporting of between-group differences with CIs, requiring imputation in some cases (10 studies). Fifth, the median follow-up of 13 weeks is relatively short. Sixth, diabetes status could not be assessed as a subgroup variable because of inconsistent reporting. Seventh, LILACS was specified in the original PROSPERO protocol but was not searched (portal inaccessible during execution); the deviation was registered as an amendment. Eighth, formal inter-rater reliability statistics were not prospectively recorded; AI-assisted classification served a triage role with all outputs verified independently by both reviewers. Ninth, the major diabetes CV outcome trials (EMPA-REG OUTCOME, CANVAS, DECLARE-TIMI 58) were excluded because their primary populations were patients with type 2 diabetes, not HF; their HF subgroup analyses were excluded as post-hoc sub-analyses. Tenth, Embase was not searched due to lack of institutional access.

Implications for Practice and Research

These findings support the continued use of SGLT2 inhibitors as a foundational therapy in HF across the LVEF spectrum, with strongest support for HFrEF mortality reduction and consistent HFH benefit across all phenotypes. Clinicians should counsel patients on genital infection risk and consider age, frailty, and concomitant therapy when initiating an SGLT2 inhibitor. The role of SGLT2 inhibitors in HFmrEF - where only three trials were available - warrants dedicated investigation. Future trials should prioritize longer follow-up to establish durability of mortality benefit, direct comparisons between SGLT2 inhibitors to assess potential within-class differences, and enrollment of underrepresented populations including patients with acute decompensated and advanced HF. The emerging agents (henagliflozin, licogliflozin) and the dual SGLT1/2 inhibitor sotagliflozin deserve further evaluation in HF-specific trials.

Data and Code Availability

The complete analysis archive - protocol with amendments, search strategies, screening and extraction pipelines, risk of bias assessments, R analysis scripts, analytic objects, and all output figures and tables - is openly deposited, and the full data-and-code availability statement, including repository identifiers and the reproducibility command, is provided in Appendix E.

## Conclusions

In adults with HF, SGLT2 inhibitors significantly reduce HF hospitalization, CV death, the composite of CV death and HF hospitalization, and serious adverse events, with moderate-to-high certainty of evidence. A reduction in all-cause mortality was also observed, but with low certainty and sensitivity to publication bias on trim-and-fill adjustment, with the strongest signal seen in HF with reduced ejection fraction. Quality-of-life improvement on the KCCQ was supported by high-certainty evidence. Among safety outcomes, only genital infections were significantly increased; no significant increases were observed for DKA, AKI, UTI, or hypotension. Benefits appeared consistent across individual SGLT2 inhibitors and across HF phenotypes, with the smaller mortality benefit in HF with preserved ejection fraction representing an evidence gap rather than a demonstrated absence of effect. The dual sodium-glucose cotransporter-1/2 inhibitor sotagliflozin showed concordant directional effects, supporting class-effect interpretation while acknowledging pharmacological distinction. These findings support the continued use of SGLT2 inhibitors as a foundational therapy across the HF spectrum, with appropriate counseling on genital infection risk. Future trials should prioritize longer follow-up to establish durability of mortality benefit, direct comparisons between agents, and enrollment of underrepresented populations including patients with acute decompensated and advanced HF.

## Appendices

Appendix A: database sensitivity rationale (LILACS and Embase)

LILACS (Latin American and Caribbean Health Sciences Literature) was specified in the original International Prospective Register of Systematic Reviews (PROSPERO) protocol but was not searched because the LILACS portal (pesquisa.bvsalud.org) returned HTTP 403 errors during the execution window in March 2026 and was inaccessible. Scoping searches conducted before LILACS became inaccessible identified no SGLT2 inhibitor heart failure RCTs published exclusively in LILACS-indexed journals not already captured by PubMed and Cochrane Central Register of Controlled Trials (CENTRAL). Cardiovascular RCTs published in Latin American journals are routinely co-indexed by these primary sources for high-impact randomized trials.

Embase was not searched due to lack of institutional access for the independent research team. Sensitivity analyses for cardiovascular meta-analyses generally suggest that PubMed plus CENTRAL plus ClinicalTrials.gov plus a major trial registry (World Health Organization International Clinical Trials Registry Platform [ICTRP]) provide acceptable coverage of large randomized cardiovascular intervention trials, with citation chasing of included studies and prior meta-analyses providing additional risk mitigation. Both deviations were registered as PROSPERO amendments.

Appendix B: screening pipeline detail

Title and abstract screening was performed using a four-tier exclusion hierarchy applied in cascade: tier 1 (animal/in vitro/non-human), tier 2 (wrong intervention - non-sodium-glucose cotransporter-2 [SGLT2]/non-sotagliflozin), tier 3 (wrong population - non-heart-failure or pediatric), tier 4 (wrong design - non-randomized, observational, single-arm, or active-comparator head-to-head). A key trial protection list (DAPA-HF, EMPEROR-Reduced, EMPEROR-Preserved, DELIVER, SOLOIST-WHF, EMPULSE, PRESERVED-HF, MUSCAT-HF, CHIEF-HF, DETERMINE, CAMEO-DAPA, CANDLE) prevented false exclusion of sentinel studies regardless of tier output. Full-text screening operated as a structured eight-pass pipeline: pass 1 (automated eligibility checks against extracted PDF metadata), pass 2 (PubMed and ClinicalTrials.gov API enrichment), pass 3 (large language model triage for likely-eligible flagging), pass 4 (trial clustering - grouping by NCT identifier, parent trial name, and sub-analysis indicator regex), pass 5 (automated FT06 exclusion for sub-analyses meeting all of: parent trial name in title, sub-analysis indicator present, not the primary publication), pass 6 (independent reviewer assessment in blocks of 25 records with verified-include / exclude / flag verdicts), pass 7 (PICO verification of full-text PDFs), pass 8 (final reconciliation against the trial cluster table). All decisions, exclusion codes, and the complete pipeline are reproducible from data files, and scripts archived on Zenodo (DOI: 10.5281/zenodo.21074013).

Appendix C: AI tool disclosure (PRISMA-AI consistent)

Two artificial intelligence tools were used during this review in non-judgmental, structuring roles: Claude (Anthropic) for initial title and abstract triage classification and for parsing full-text PDF content into a standardized long-format extraction structure, and Codex (OpenAI) for assistance with extraction structuring and helper script generation. AI-assisted classification labels and AI-extracted values were treated as candidate inputs requiring independent human verification; both reviewers (VMF, VAM) verified each AI output against the source publication before any value was admitted to the analysis dataset. AI tools were not used for risk of bias assessment, statistical analysis, Grading of Recommendations Assessment, Development and Evaluation (GRADE) certainty rating, or interpretation of results. Formal validation metrics for the AI triage step (sensitivity, specificity, proportion of extracted values requiring correction) were not prospectively recorded and represent a limitation. AI was not used during manuscript drafting beyond editorial assistance.

Appendix D: imputation methods' details

Continuous outcomes occasionally required imputation when between-group differences were not directly reported with confidence intervals. Standard deviations were derived from standard errors using SD = SE × sqrt(n) and from confidence intervals using SD = (CI_upper − CI_lower) × sqrt(n) / (2 × t-critical). When studies reported medians with interquartile ranges, the Wan et al. (2014) estimator was applied. When repeated-measures change scores were reported as separate baseline and follow-up means without baseline-follow-up correlation, a conservative correlation of 0.5 was assumed. All imputations were documented per study and outcome; the sensitivity analysis excluding studies with imputed data is reported in the Findings section.

Appendix E: data and code availability

The complete analysis archive - including the protocol with all amendments, search strategies for each database, screening pipeline scripts, deduplication audit trail, full-text screening decisions and reasons, data extraction form (long and wide formats), risk of bias assessments, R analysis scripts, RDS analytic objects, all output figures and tables, and rendered manuscript files - is deposited on Zenodo (DOI: 10.5281/zenodo.21074013; https://doi.org/10.5281/zenodo.21074013). The PROSPERO record (CRD420251167908) is available at https://www.crd.york.ac.uk/prospero/. All five PROSPERO amendments are documented in the local protocol/amendments_log.md file within the Zenodo archive.

Reproducibility command:

Rscript scripts/R/run_all.R

This regenerates every figure, table, and analytic object from the data files committed to the archive.
